# Supplementary material for: Evaluation of Antibody Response to Heterologous Prime–Boost Vaccination with ChAdOx1 nCoV-19 and BNT162b2: An Observational Study
Source: Vaccines (Basel). 2021 Dec 14;9(12):1478. doi: 10.3390/vaccines9121478 (PMC8704060; doi:10.3390/vaccines9121478)
Supplement: Supplementary file 1 [file vaccines-09-01478-s001.zip › vaccines-1484710-supplementary.pdf]

Supplementary Table S1: The IgG anti-S-RBD levels of study groups assessed 4 weeks after vaccine booster, according to gender and vaccination schedule of enrolled subjects.

Gender			**BNT/BNT** n=50	**ChAd/ChAd** n=36	**ChAd/BNT** n=49
Male	Median		361.60	19.89	379.25
	Minimum		43.42	4.51	66.15
	Maximum		1264.90	69.62	1000.00
	Percentile	25	158.10	11.87	133.87
		50	361.60	19.89	379.25
		75	538.85	40.69	1000.00
Female	Median		238.50	52.74	551.20
	Minimum		88.37	1.90	43.29
	Maximum		1014.00	555.00	1000.00
	Percentile	25	174.50	18.52	235.50
		50	238.50	52.74	551.20
		75	437.70	82.37	1000.00

BNT/BNT: two doses of BNT162b2, 3 weeks apart.

ChAd/ChAd: two doses of ChAdOx1 nCoV-19, 8 to 12 weeks apart.

ChAd/BNT: first dose of ChAdOx1 nCoV-19, second dose BNT162b2, 8 to 12 weeks apart.

Supplementary Table S2: The IgG anti-S-RBD levels of study subjects assessed 15 weeks after vaccine booster, according to gender and vaccination schedule of enrolled subjects.

Gender			**BNT/BNT** n=15	**ChAd/ChAd** n=9	**ChAd/BNT** n=17
Male	Median		60.60	n.a.	84.91
	Minimum		27.00	n.a.	30.00
	Maximum		99.00	n.a.	183.00
	Percentile	25	28.00	n.a.	39.95
		50	60.60	n.a.	84.91
		75	96.80	n.a.	162.25
Female	Median		90.00	19.74	400.00
	Minimum		20.00	3.06	110.00
	Maximum		247.00	100.00	580.00
	Percentile	25	49.00	5.57	235.00
		50	90.00	19.74	400.00
		75	188.00	42.12	450.00

BNT/BNT: two doses of BNT162b2, 3 weeks apart.

ChAd/ChAd: two doses of ChAdOx1 nCoV-19, 8 to 12 weeks apart.

ChAd/BNT: first dose of ChAdOx1 nCoV-19, second dose BNT162b2, 8 to 12 weeks apart.
